# Supplementary material for: APOE ε2 is associated with increased tau pathology in primary tauopathy
Source: Nat Commun. 2018 Oct 22;9:4388. doi: 10.1038/s41467-018-06783-0 (PMC6197187; doi:10.1038/s41467-018-06783-0)
Supplement: Supplementary file 1 — Supplementary Information [file 41467_2018_6783_MOESM1_ESM.pdf]

Supplementary information

APOE ε2 is associated with increased tau pathology in primary tauopathy

Zhao et al.

Inventory

Supplementary Figures 1-11

Supplementary Figure 1: The gene copy numbers and expression levels of human tau in the brain of Tau^{P301L}-apoE mice.
Supplementary Figure 2: Widespread expression of human tau protein in the brain of Tau^{P301L}-apoE mice.
Supplementary Figure 3: Hyperphosphorylated tau species, Thioflavin S-positive tau aggregates, and astrogliosis in the brain of Tau^{P301L}-apoE mice.
Supplementary Figure 4: Accumulation of hyperphosphorylated tau species in the brain of Tau^{P301L}-apoE mice.
Supplementary Figure 5: The examination of neuronal loss in Tau^{P301L}-apoE mice at 6 months of age by Nissl staining.
Supplementary Figure 6: The examination of neuronal loss in Tau^{P301L}-apoE mice at 6 months of age by NeuN staining.
Supplementary Figure 7: No prominent microgliosis and microglial activation in Tau^{P301L}-apoE mice.
Supplementary Figure 8: Behavioral abnormalities in Tau^{P301L}-apoE mice.
Supplementary Figure 9: ApoE mRNA expression in Tau^{P301L}-apoE mice.
Supplementary Figure 10: Disulfide bond-mediated complex formation between tau and apoE lipoprotein particles *in vitro*.
Supplementary Figure 11: Uncropped scans of blots shown in Fig. 2e, Fig. 3a and e, Fig. 4a, and Supplementary Fig. 10.

Supplementary Tables 1-5

Supplementary Table 1: Characteristics of the PSP patients.
Supplementary Tables 2a-c: Summary of semi-quantitative tau pathology measures CB, NFT, TA, NT in 20 brain regions.
Supplementary Table 3: Association between *APOE* ε2 and semi-quantitative tau pathology severity scores.
Supplementary Table 4: Association of *APOE* genotype with Thal amyloid phase and TDP-43 pathology.
Supplemental Table 5: Subject characteristics for human genetic study.

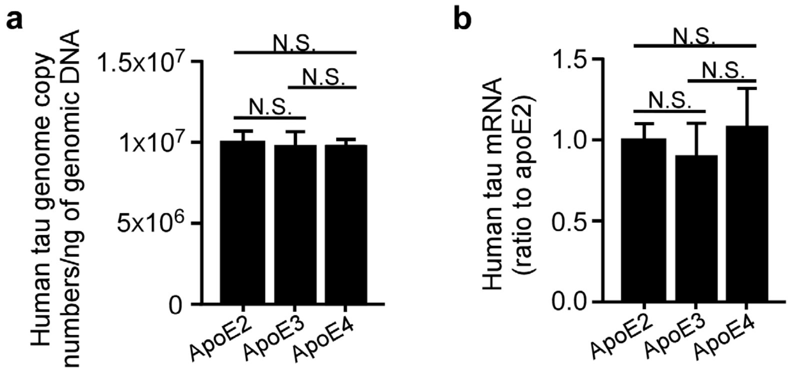

Supplementary Figure 1: The gene copy numbers and expression levels of human tau in the brain of Tau^{P301L}-apoE mice. AAV-Tau^{P301L} genome copy numbers and tau mRNA levels were determined in the cortex of Tau^{P301L}-apoE2, -apoE3, and -apoE4 mice at 6 months of age (n=8 mice/group, mixed gender). (a) Genomic DNA was isolated from cortex and the vector genome copy numbers/ng of genomic DNA was determined using qPCR. (b) RNA was isolated from cortex and human tau mRNA expression was detected by qPCR. Data are expressed as mean ± SEM. Mann-Whitney tests followed by Bonferroni correction for multiple comparisons were used. P-values < 0.0167 were considered to be statistically significant. N.S., not significant.

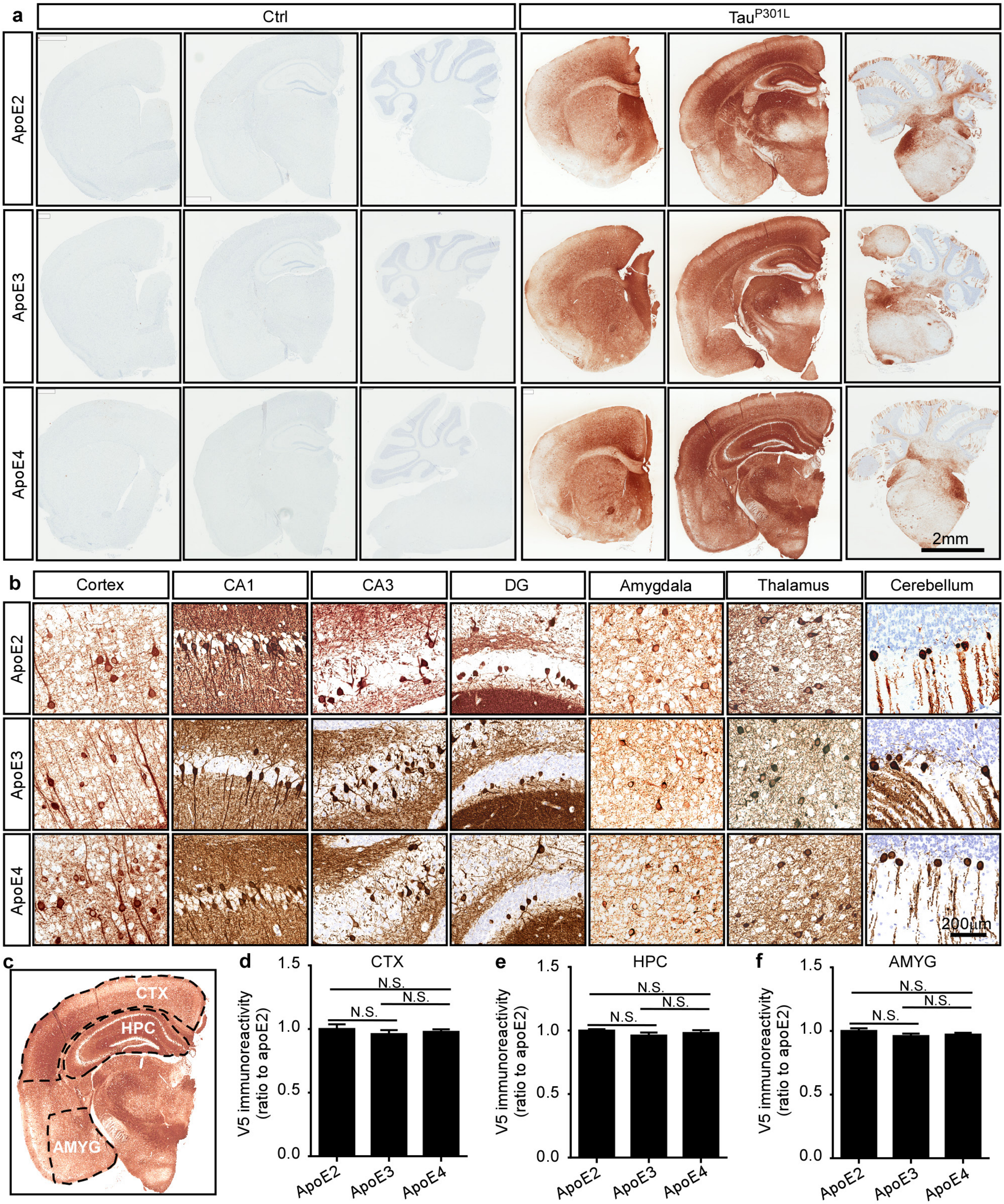

Supplementary Figure 2: Widespread expression of human tau protein in the brain of Tau^{P301L}-apoE mice.

Brain slices from control (Ctrl) and V5-tagged Tau^{P301L}-apoE2, -apoE3, and -apoE4 mice at 6 months of age were stained with V5 antibody whose immunoreactivity represents the expression of human tau. (a) The V5 expression in three different brain planes of the coronal sections from apoE-TR mice expressing GFP or human Tau^{P301L} was shown. Scale bar, 2mm. (b) The V5 expression pattern in the region of cortex, CA1, CA3, DG, amygdala, thalamus and cerebellum from Tau^{P301L}-apoE mice was shown. Scale bar, 200 µm. (c-f) The immunoreactivity of V5 staining in the cortex (CTX, d), hippocampus (HPC, e), and amygdala (AMYG, f) from Tau^{P301L}-apoE mice was quantified using Aperio ImageScope (n=12 mice/group in Ctrl groups, n=13 mice for Tau^{P301L}-apoE2 group, n=20 mice for Tau^{P301L}-apoE3 group, n=22 mice for Tau^{P301L}-apoE4 group, mixed gender). The boundaries of the relevant regions were shown (c). Data are expressed as mean ± SEM. Mann-Whitney tests followed by Bonferroni correction for multiple comparisons were used. P-values < 0.0167 were considered to be statistically significant. N.S., not significant.

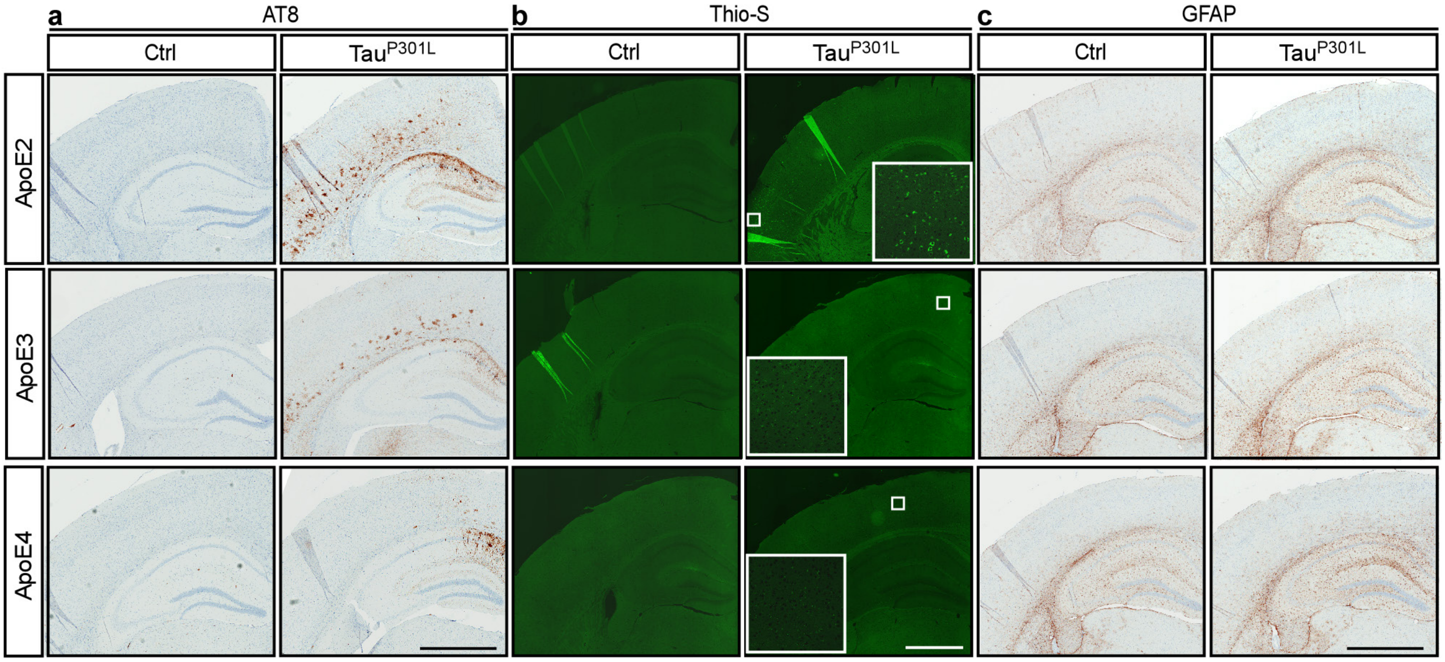

Supplementary Figure 3: Hyperphosphorylated tau species, Thioflavin S-positive tau aggregates, and astrogliosis in the brain of Tau^{P301L}-apoE mice.

The hyperphophorylated tau, aggregated tau, and astrogliosis were determined by AT8, Thioflavin S, and GFAP staining in the brain slices from AAV-GFP control mice (Ctrl) and AAV-Tau^{P301L}-apoE2, -apoE3, and -apoE4 mice at 6 months of age. Representative images are shown for the deposition of AT8-positive tau species (a, scale bar 1mm), Thioflavin S-positive tau aggregates (b, scale bar 800 µm), and GFAP-positive astrogliosis (c, scale bar 1mm).

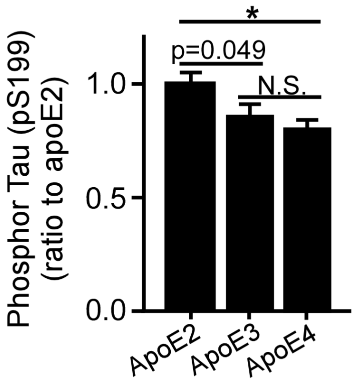

Supplementary Figure 4: Accumulation of hyperphosphorylated tau species in the brain of Tau^{P301L}-apoE mice.

The amount of hyperphosphorylated tau (pS199) in the cortex (RIPA-soluble fraction) of control and Tau^{P301L}-apoE mice (n=8 mice/group, mixed gender) at 6 months of age was examined by ELISA. Data are expressed as mean ± SEM. Mann-Whitney tests followed by Bonferroni correction for multiple comparisons were used. *$p < 0.0167$; N.S., not significant.

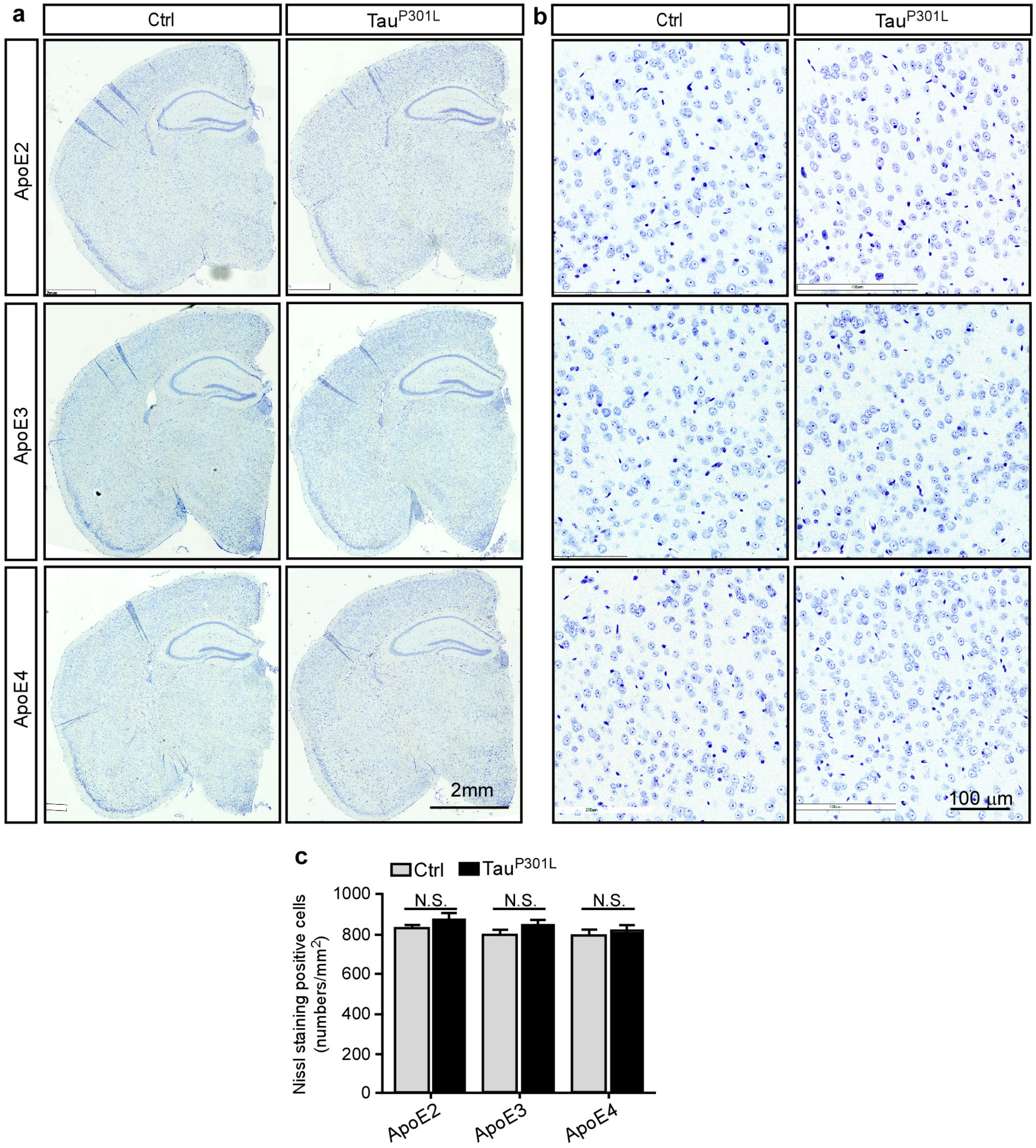

Supplementary Figure 5: The examination of neuronal loss in Tau^{P301L}-apoE mice at 6 months of age by Nissl staining. Brain slices from control (Ctrl) and Tau^{P301L}-apoE2, -apoE3, and -apoE4 mice at 6 months of age were performed by Nissl staining (a, scale bar is 2 mm). Representative images were shown for the Nissl staining-positive cells in the cortex of control or Tau^{P301L}-apoE2, -apoE3, and -apoE4 mice at 6 months of age (b, scale bar is 100 μm). The numbers of Nissl staining-positive cells in the cortical region were quantified by Aperio ImageScope (n=6 mice/group, mixed gender) (c). Data represent mean ± SEM. Mann-Whitney tests followed by Bonferroni correction for multiple comparisons were used. P-values < 0.0167 were considered to be statistically significant. N.S., not significant.

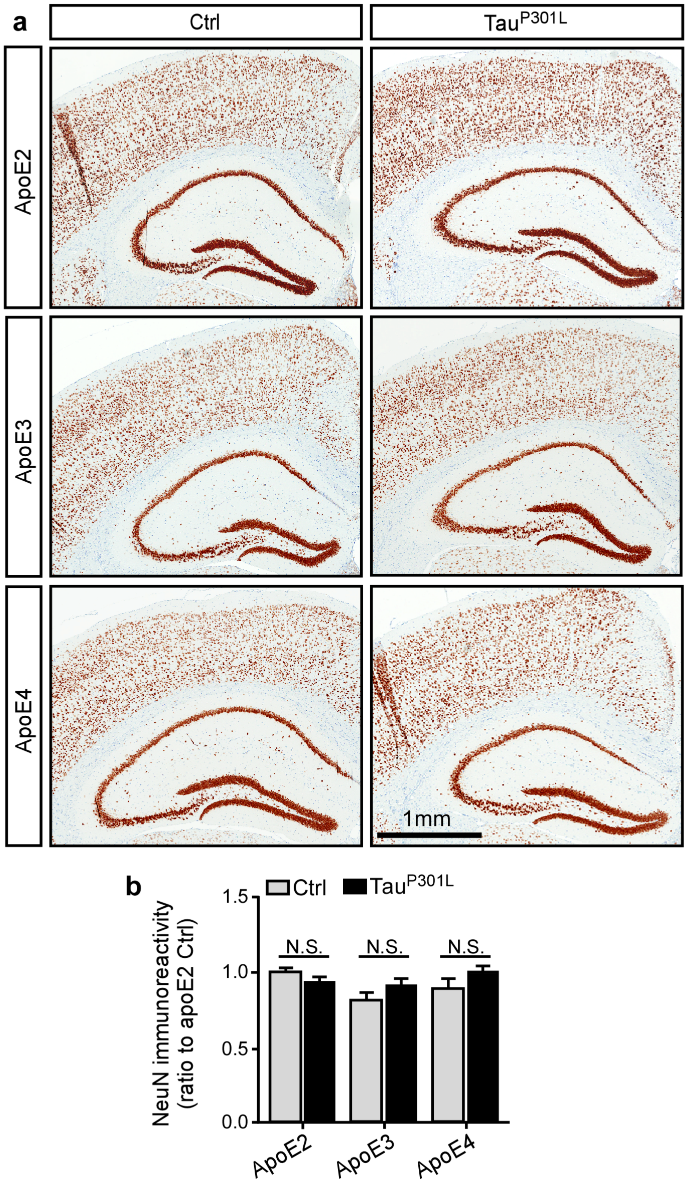

Supplementary Figure 6: The examination of neuronal loss in Tau^{P301L}-apoE mice at 6 months of age by NeuN staining. Brain slices from control (Ctrl) and Tau^{P301L}-apoE2, -apoE3, and -apoE4 mice at 6 months of age were immunostained by anti-NeuN antibody. Representative images were shown for the NeuN-positive cells in the cortex of control or Tau^{P301L}-apoE2, -apoE3, and -apoE4 mice at 6 months of age (a). The immunoreactivity of NeuN staining was evaluated by Aperio ImageScope (n=6 mice/group, mixed gender) (b). Data represent mean ± SEM. Mann-Whitney tests followed by Bonferroni correction for multiple comparisons were used. P-values < 0.0167 were considered to be statistically significant. N.S., not significant. Scale bar, 1 mm.

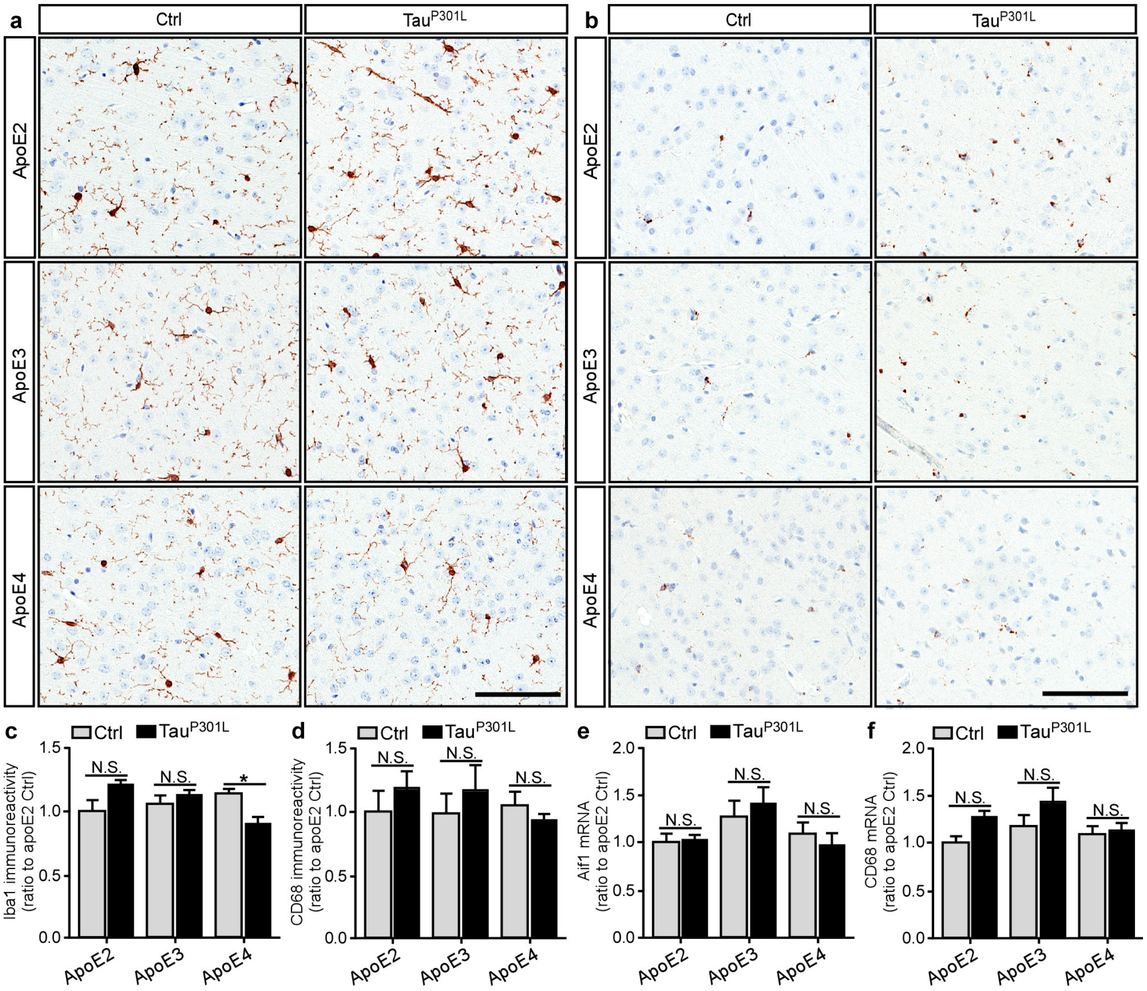

Supplementary Figure 7: No prominent microgliosis and microglial activation in Tau^{P301L}-apoE mice.

Brain slices from control (Ctrl) and Tau^{P301L}-apoE2, -apoE3, and -apoE4 mice at 6 months of age were immunostained by anti-Iba1 or anti-CD68 antibody. Representative images were shown for the Iba1-positive (a) or CD68-positive (b) microglia in the cortex of control or Tau^{P301L}-apoE2, -apoE3, and -apoE4 mice at 6 months of age (a). The immunoreactivity of Iba1 (c) and CD68 (d) staining was evaluated by Aperio ImageScope (n=6 mice/group, mixed gender). RNA was isolated from brain cortex of Ctrl and Tau^{P301L}-apoE2, -apoE3, and -apoE4 mice at 6 months of age (n=8 mice/group, mixed gender), and the *Aif1* (e) and *CD68* (f) mRNA levels were detected with qPCR. Data represent mean ± SEM. Mann-Whitney tests followed by Bonferroni correction for multiple comparisons were used. *$p < 0.0167$; N.S., not significant. Scale bar, 100 μm.

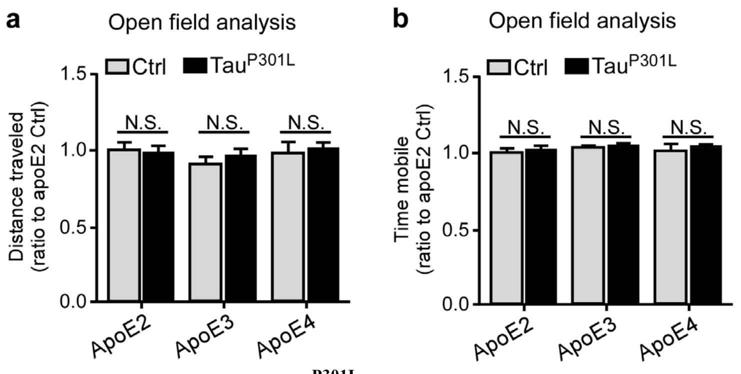

Supplementary Figure 8: Behavioral abnormalities in Tau^{P301L}-apoE mice.

Anxiety-related behaviors were analyzed in control (Ctrl, n=12 mice/group, mixed gender) and Tau^{P301L}-apoE2, -apoE3, and -apoE4 mice (n=13 mice for Tau^{P301L}-apoE2 group, n=20 mice for Tau^{P301L}-apoE3 group, n=22 mice for Tau^{P301L}-apoE4 group, mixed gender) assessed by open field analysis at 6 months of age. The total distance traveled (a), and time spent mobile (b) were shown. Data represent mean ± SEM. Mann-Whitney tests followed by Bonferroni correction for multiple comparisons were used. P-values < 0.0167 were considered to be statistically significant. N.S., not significant.

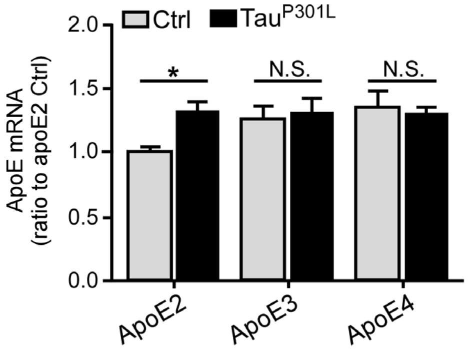

Supplementary Figure 9: ApoE mRNA expression in Tau^{P301L}-apoE mice.

RNA was isolated from brain cortex of the control (Ctrl) and Tau^{P301L}-apoE2, -apoE3, and -apoE4 mice at 6 months of age (n=8 mice/group, mixed gender). ApoE mRNA expression was detected with qPCR. Data are expressed as mean ± SEM. Mann-Whitney tests followed by Bonferroni correction for multiple comparisons were used. *p < 0.0167; N.S., not significant.

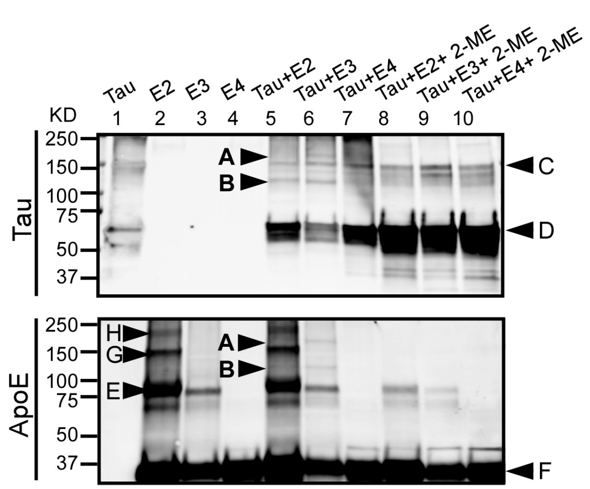

Supplementary Figure 10: Disulfide bond-mediated complex formation between tau and apoE lipoprotein particles *in vitro*.

The interaction between tau and lipidated-apoE particle was evaluated by solution binding assay, followed by Western blotting (three independent experiments). Recombinant human tau protein and immortalized astrocyte secreted apoE particles were incubated for 1 hr at 37°C in 20 µl of phosphate-buffered saline. The reaction was quenched by addition of 20 µl of sample buffer without (Lane 1-7) or with (Lane 8-10) 2-mercaptoethanol (2-ME). The tau/apoE complexes (bands A and B) were determined by both tau and apoE immunoblotting. Band C was a nonspecific band from tau immunoblot. Bands D and F were monomeric tau and apoE, respectively. Bands E, G, and H were estimated to be apoE dimer, tetramer, and hexamer, respectively.

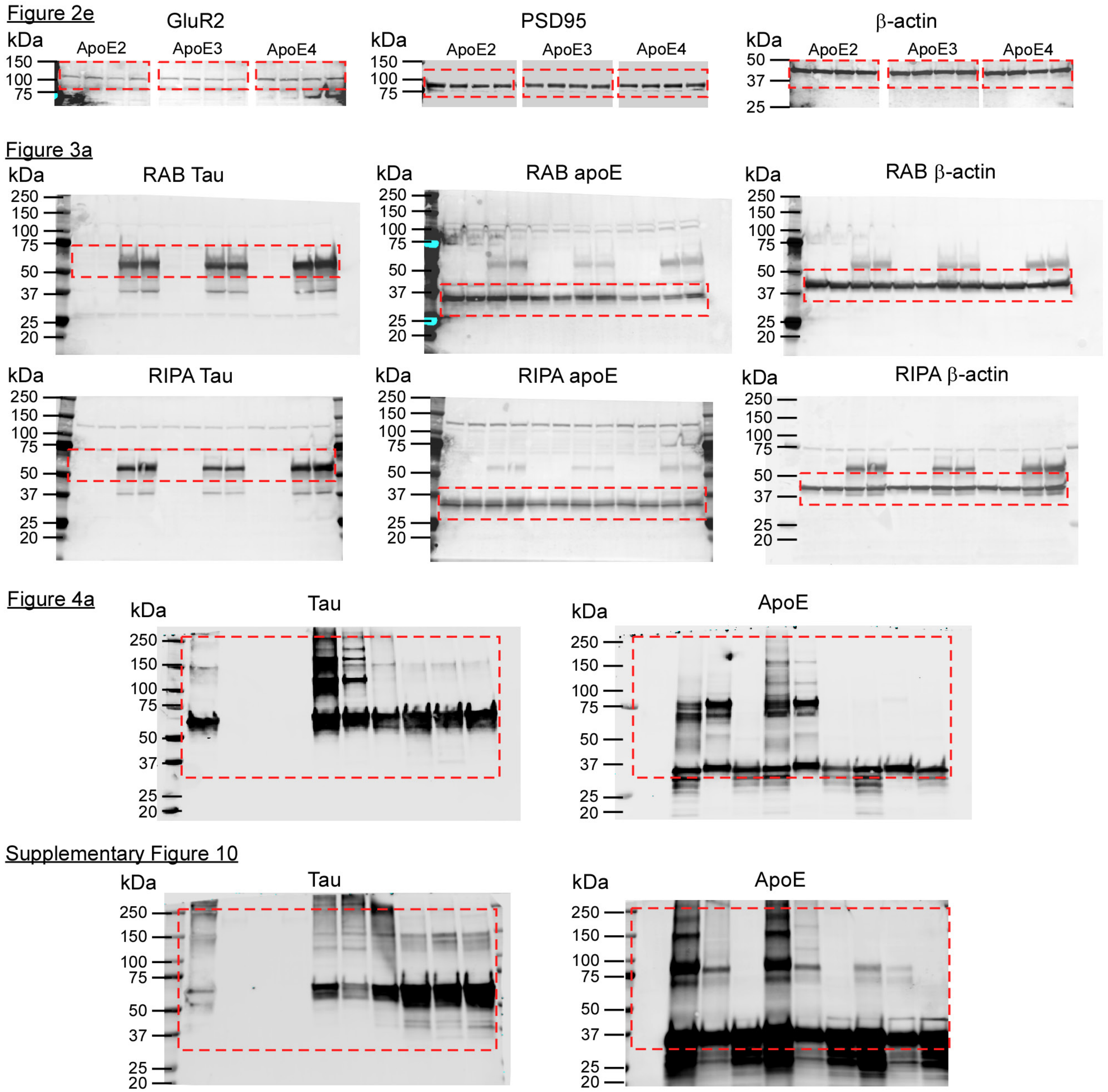

Supplementary Figure 11: Uncropped scans of blots shown in Fig. 2e, Fig. 3a and e, Fig. 4a, and Supplementary Fig. 10.

Supplemental Table 1: Characteristics of PSP patients for human pathological study.

Variable	Summary (N=858)
Age at death (years)	75 (52, 98)
Sex	
Female	399 (46%)
Male	459 (54%)
Braak NFT stage	
0	120 (14%)
I	142 (17%)
II	253 (30%)
III	270 (32%)
IV	54 (6%)
V	12 (1%)
VI	7 (0.8%)
Thal amyloid phase	
0	377 (44%)
1	147 (17%)
2	61 (7%)
3	202 (24%)
4	47 (6%)
5	24 (3%)
TDP-43 pathology (presence)	36 (5%)
APOE genotype	
ε2/ε2	12 (1.4%)
ε2/ε3	98 (11%)
ε2/ε4	19 (2%)
ε3/ε3	549 (64%)
ε3/ε4	168 (20%)
ε4/ε4	12 (1.4%)
CB overall tau pathology score	1.6 (0.2, 2.5)
NFT overall tau pathology score	2.2 (0.8, 2.9)
TA overall tau pathology score	1.0 (0.1, 2.4)
NT overall tau pathology score	2.2 (0.3, 2.9)

The sample median (minimum, maximum) is given for continuous variables. Information was unavailable regarding TDP-43 pathology for 94 patients.

Supplemental Table 2a: Summary of semi-quantitative tau pathology measures CB, NFT, TA, NT in 20 brain regions.

Anatomical region	CB	NFT	TA	NT
Basal nucleus of Meynert				
Missing	21	1	43	4
0=None	214 (26%)	2 (0.2%)	434 (53%)	44 (5%)
1=Mild	552 (66%)	27 (3%)	333 (41%)	183 (21%)
2=Moderate	67 (8%)	277 (32%)	47 (6%)	333 (39%)
3=Severe	4 (0.5%)	551 (64%)	1 (0.1%)	294 (34%)
Caudate nucleus				
Missing	1	1	1	13
0=None	28 (3%)	1 (0.1%)	5 (0.6%)	52 (6%)
1=Mild	444 (528%)	295 (34%)	65 (8%)	506 (60%)
2=Moderate	271 (32%)	491 (57%)	201 (24%)	253 (30%)
3=Severe	114 (13%)	70 (8%)	586 (68%)	34 (4%)
Globus pallidus				
Missing	2	1	39	8
0=None	17 (2%)	3 (0.4%)	307 (38%)	43 (5%)
1=Mild	169 (20%)	155 (18%)	327 (40%)	191 (22%)
2=Moderate	307 (36%)	485 (57%)	165 (20%)	378 (44%)
3=Severe	363 (42%)	214 (25%)	20 (2%)	238 (28%)
Hypothalamus				
Missing	81	20	88	27
0=None	454 (58%)	0 (0%)	616 (80%)	89 (11%)
1=Mild	309 (40%)	48 (6%)	142 (18%)	382 (46%)
2=Moderate	13 (2%)	270 (32%)	7 (1%)	252 (30%)
3=Severe	1 (0.1%)	520 (62%)	5 (1%)	108 (13%)
Motor cortex				
Missing	9	5	7	16
0=None	25 (3%)	10 (1%)	17 (2%)	59 (7%)
1=Mild	170 (20%)	206 (24%)	134 (16%)	196 (23%)
2=Moderate	396 (47%)	446 (52%)	207 (24%)	319 (38%)
3=Severe	258 (30%)	191 (22%)	493 (58%)	268 (32%)
Subthalamic nucleus				
Missing	2	2	18	4
0=None	4 (0.5%)	1 (0.1%)	182 (22%)	4 (0.5%)
1=Mild	169 (20%)	68 (8%)	225 (27%)	44 (5%)
2=Moderate	358 (42%)	270 (32%)	220 (26%)	109 (13%)
3=Severe	325 (38%)	517 (60%)	213 (25%)	697 (82%)
Inferior temporal gyrus				
Missing	62	21	41	85
0=None	493 (62%)	168 (20%)	204 (25%)	573 (74%)
1=Mild	270 (34%)	517 (62%)	487 (60%)	107 (14%)
2=Moderate	30 (4%)	102 (12%)	108 (13%)	34 (4%)
3=Severe	3 (0.4%)	50 (6%)	18 (2%)	59 (8%)

Supplemental Table 2b: Summary of semi-quantitative tau pathology measures CB, NFT, TA, NT in 20 brain regions.

Anatomical region	CB	NFT	TA	NT
Thalamic fasciculus				
Missing	2	N/A	N/A	3
0=None	2 (0.2%)	N/A	N/A	6 (0.7%)
1=Mild	85 (10%)	N/A	N/A	90 (10%)
2=Moderate	293 (34%)	N/A	N/A	319 (37%)
3=Severe	476 (56%)	N/A	N/A	440 (52%)
Ventral thalamus				
Missing	1	1	15	2
0=None	8 (1%)	5 (1%)	93 (11%)	11 (1%)
1=Mild	137 (16%)	169 (20%)	321 (38%)	49 (6%)
2=Moderate	301 (35%)	457 (53%)	316 (38%)	221 (26%)
3=Severe	411 (48%)	226 (26%)	113 (13%)	575 (67%)
Cerebellar white matter				
Missing	6	N/A	N/A	12
0=None	22 (3%)	N/A	N/A	69 (8%)
1=Mild	178 (21%)	N/A	N/A	363 (43%)
2=Moderate	393 (46%)	N/A	N/A	263 (31%)
3=Severe	259 (30%)	N/A	N/A	151 (18%)
Dentate nucleus				
Missing	33	11	86	10
0=None	216 (26%)	6 (1%)	662 (86%)	20 (2%)
1=Mild	538 (65%)	123 (14%)	99 (13%)	276 (32%)
2=Moderate	69 (8%)	339 (40%)	10 (1%)	494 (58%)
3=Severe	2 (0.2%)	379 (45%)	1 (0.1%)	58 (7%)
Inferior olivary nucleus				
Missing	66	61	99	58
0=None	103 (13%)	30 (4%)	542 (71%)	13 (2%)
1=Mild	536 (68%)	331 (42%)	192 (25%)	64 (8%)
2=Moderate	142 (18%)	312 (39%)	24 (3%)	187 (23%)
3=Severe	11 (1%)	124 (16%)	1 (0.1%)	536 (67%)
Locus ceruleus				
Missing	141	42	N/A	43
0=None	638 (89%)	1 (0.1%)	N/A	20 (2%)
1=Mild	70 (10%)	32 (4%)	N/A	119 (15%)
2=Moderate	7 (1%)	225 (28%)	N/A	350 (43%)
3=Severe	2 (0.3%)	558 (68%)	N/A	326 (40%)
Medullary tegmentum				
Missing	50	44	134	47
0=None	80 (10%)	2 (0.2%)	643 (89%)	5 (1%)
1=Mild	428 (53%)	15 (2%)	76 (10%)	38 (5%)
2=Moderate	259 (32%)	188 (23%)	4 (1%)	111 (14%)
3=Severe	41 (5%)	609 (75%)	1 (0.1%)	657 (81%)

N/A is given when the given lesion was not assessed in the given anatomical structure.

Supplemental Table 2c: Summary of semi-quantitative tau pathology measures CB, NFT, TA, NT in 20 brain regions.

Anatomical region	CB	NFT	TA	NT
Midbrain tectum				
Missing	6	20	23	7
0=None	14 (2%)	2 (0.2%)	44 (5%)	8 (1%)
1=Mild	93 (11%)	194 (23%)	150 (18%)	45 (5%)
2=Moderate	279 (33%)	424 (51%)	337 (40%)	130 (15%)
3=Severe	466 (55%)	218 (26%)	304 (36%)	668 (78%)
Oculomotor complex				
Missing	158	116	189	117
0=None	329 (47%)	5 (1%)	546 (82%)	36 (5%)
1=Mild	308 (44%)	146 (20%)	106 (16%)	178 (24%)
2=Moderate	54 (8%)	297 (40%)	16 (2%)	281 (38%)
3=Severe	9 (1%)	294 (40%)	1 (0.1%)	246 (33%)
Pontine base				
Missing	15	6	103	14
0=None	103 (12%)	14 (2%)	682 (90%)	59 (7%)
1=Mild	524 (62%)	98 (12%)	67 (9%)	224 (26%)
2=Moderate	204 (24%)	182 (21%)	6 (1%)	392 (46%)
3=Severe	12 (1%)	558 (66%)	0 (0%)	169 (20%)
Pontine tegmentum				
Missing	28	10	94	24
0=None	27 (3%)	1 (0.1%)	625 (82%)	7 (1%)
1=Mild	277 (33%)	92 (11%)	124 (16%)	55 (7%)
2=Moderate	371 (45%)	342 (40%)	13 (2%)	182 (22%)
3=Severe	155 (19%)	413 (49%)	2 (0.3%)	590 (71%)
Red nucleus				
Missing	6	4	21	4
0=None	23 (3%)	9 (1%)	145 (17%)	17 (2%)
1=Mild	130 (15%)	202 (24%)	281 (34%)	81 (10%)
2=Moderate	234 (28%)	398 (47%)	276 (33%)	198 (23%)
3=Severe	465 (55%)	245 (29%)	135 (16%)	558 (65%)
Substantia nigra				
Missing	6	3	31	3
0=None	50 (6%)	0 (0%)	354 (43%)	3 (0.4%)
1=Mild	422 (50%)	99 (12%)	316 (38%)	194 (23%)
2=Moderate	309 (36%)	404 (47%)	140 (17%)	460 (54%)
3=Severe	71 (8%)	352 (41%)	17 (2%)	198 (23%)

Supplemental Table 3: Association between *APOE* ε2 and semi-quantitative tau pathology severity scores.

	Association between presence of *APOE* ε2 (vs. ε3/ε3) and CB		Association between presence of *APOE* ε2 (vs. ε3/ε3) and TA		Association between presence of *APOE* ε2 (vs. ε3/ε3) and NT	
**Anatomical region**	**OR (95% CI)**	**P-Value**	**OR (95% CI)**	**P-Value**	**OR (95% CI)**	**P-Value**
Basal nucleus	1.48 (0.98, 2.24)	0.066	1.92 (1.31, 2.80)	0.001	1.59 (1.11, 2.28)	0.012
Caudate putamen	1.18 (0.81, 1.70)	0.39	1.31 (0.86, 2.01)	0.21	1.14 (0.77, 1.67)	0.52
Globus pallidus	1.29 (0.89, 1.87)	0.18	1.64 (1.14, 2.37)	0.008	1.05 (0.73, 1.51)	0.78
Hypothalamus	1.02 (0.68, 1.53)	0.92	1.26 (0.78, 2.03)	0.36	1.00 (0.70, 1.44)	0.98
Motor cortex	1.05 (0.73, 1.50)	0.81	1.33 (0.90, 1.97)	0.15	1.19 (0.84, 1.70)	0.33
Subthalamic nucleus	1.36 (0.94, 1.95)	0.10	1.65 (1.16, 2.34)	0.005	2.05 (1.13, 3.70)	0.018
Temporal cortex	1.24 (0.82, 1.87)	0.31	1.15 (0.78, 1.71)	0.48	1.12 (0.67, 1.88)	0.66
Thalamic fasciculus	1.18 (0.80, 1.74)	0.41	N/A	N/A	1.23 (0.84, 1.79)	0.29
Ventral thalamus	1.38 (0.95, 2.02)	0.095	1.52 (1.06, 2.17)	0.023	1.54 (0.99, 2.39)	0.058
Cerebellar white matter	1.15 (0.80, 1.66)	0.44	N/A	N/A	1.12 (0.78, 1.59)	0.55
Dentate nucleus	1.16 (0.77, 1.74)	0.48	1.37 (0.79, 2.36)	0.26	1.36 (0.92, 2.01)	0.12
Inferior olive	1.87 (1.24, 2.83)	0.003	1.07 (0.69, 1.67)	0.77	1.27 (0.83, 1.96)	0.27
Locus ceruleus	1.05 (0.54, 2.07)	0.88	N/A	N/A	1.37 (0.94, 1.98)	0.10
Medullary tegmentum	1.40 (0.96, 2.05)	0.080	1.01 (0.53, 1.94)	0.97	2.02 (1.10, 3.69)	0.023
Midbrain tectum	1.40 (0.95, 2.07)	0.087	1.89 (1.31, 2.73)	0.001	1.42 (0.86, 2.34)	0.17
Oculomotor complex	0.75 (0.50, 1.15)	0.19	0.57 (0.30, 1.09)	0.090	1.18 (0.80, 1.73)	0.41
Pontine base	1.47 (1.00, 2.16)	0.050	1.13 (0.58, 2.21)	0.72	1.06 (0.74, 1.51)	0.75
Pontine tegmentum	1.20 (0.84, 1.73)	0.32	1.76 (1.07, 2.90)	0.027	1.20 (0.77, 1.88)	0.42
Red nucleus	1.11 (0.76, 1.62)	0.60	1.24 (0.88, 1.76)	0.22	1.43 (0.94, 2.18)	0.098
Substantia nigra	1.30 (0.90, 1.89)	0.16	1.36 (0.94, 1.95)	0.099	1.54 (1.06, 2.22)	0.022

OR=odds ratio; CI=confidence interval. ORs, 95% CIs, and p-values result from proportional odds logistic regression models that were adjusted for age at death, sex, Braak NFT stage, and Thal amyloid phase, where the outcome measure was the semi-quantitative tau pathology measure (0=none, 1=mild, 2=moderate, 3=severe) for the given anatomical structure. ORs are interpreted as the multiplicative increase in the odds of a more severe tau pathology corresponding to the presence of the *APOE* ε2 allele (in comparison to the ε3/ε3 reference genotype). For categories of tau pathology severity measures that had fewer than 10 patients for a given anatomical structure, these were collapsed with more common categories in proportional odds regression analysis. N/A is given when the given lesion was not assessed in the given anatomical structure.

Supplemental Table 4: Association of *APOE* genotype with Thal amyloid phase and TDP-43 pathology.

	Association with Thal amyloid phase		Association with TDP-43 pathology	
APOE genotype (comparisons made vs. the ε3/ε3 reference genotype)	**OR (95% CI)**	**P-value**	**OR (95% CI)**	**P-value**
Presence of ε2	0.80 (0.56, 1.01)	0.24	1.48 (0.56, 3.94)	0.43
Presence of ε4	5.62 (4.07, 7.77)	<0.0001	1.42 (0.58, 3.51)	0.45
Presence of ε2/ε2	0.77 (0.25, 2.41)	0.65	2.58 (0.24, 27.56)	0.43
Presence of ε2/ε3	0.74 (0.55, 0.99)	0.003	1.02 (0.28, 3.66)	0.98
Presence of ε2/ε4	6.05 (2.56, 14.32)	<0.0001	2.47 (0.46, 13.33)	0.29
Presence of ε3/ε4	5.36 (3.81, 7.53)	<0.0001	1.40 (0.54, 3.65)	0.49
Presence of ε4/ε4	16.44 (5.51, 49.03)	<0.0001	NA	1.00[1]

OR=odds ratio; CI=confidence interval. For associations with Thal phase, ORs, 95% CIs, and p-values result from proportional odds logistic regression models that were adjusted for age at death, sex, and Braak stage. ORs are interpreted as the multiplicative increase in the odds of a higher Thal phase corresponding to the presence of the given *APOE* variable (in comparison to the ε3/ε3 reference genotype).. For associations with presence of TDP-43 pathology, ORs, 95% CIs, and p-values result from binary logistic regression models that were adjusted for age at death, sex, Braak stage, and Thal phase. ORs are interpreted as the multiplicative increase in the odds of presence of TDP-43 pathology corresponding to the presence of the given *APOE* variable (in comparison to the ε3/ε3 reference genotype)..[1] P-value results from Fisher's exact test due to the absence of ε4/ε4 patients with TDP-43 pathology.

Supplemental Table 5: Subject characteristics for human genetic study

**Series**	**PSP patients (N=994)**	**CBD patients (N=134)**	**Controls (N=1406)**
Age	75 (52, 98)	69 (46, 96)	75 (45, 100)
Gender			
Male	534 (53.7%)	68 (50.8%)	624 (44.4%)
Female	460 (46.3%)	66 (49.3%)	782 (55.6%)
MAPT genotype			
H1/H1	884 (89.0%)	114 (87.0%)	805 (57.3%)
H1/H2	106 (10.7%)	16 (12.2%)	514 (36.6%)
H2/H2	3 (0.3%)	1 (0.8%)	85 (6.1%)

The sample median (minimum, maximum) is given for age, which is the age at death in PSP and CBD patients and the age at blood draw in controls. *MAPT* genotype was unavailable for 1 PSP patient, 3 CBD patients, and 2 controls.
